# Two new species of *Microlicia* D.Don (Melastomataceae, Microlicieae) from Chapada dos Veadeiros, Goiás State, Brazil

**DOI:** 10.3897/phytokeys.164.57569

**Published:** 2020-10-21

**Authors:** Jean Correa Fontelas, Rosana Romero

**Affiliations:** 1 Programa de Pós-Graduação em Biologia Vegetal, Instituto de Biologia, Universidade Federal de Uberlândia, Rua Ceará, s.n., 38400-902, Uberlândia, Minas Gerais, Brazil Universidade Federal de Uberlândia Uberlândia Brazil; 2 Instituto de Biologia, Universidade Federal de Uberlândia, Rua Ceará, s.n., 38400-902, Uberlândia, Minas Gerais, Brazil Universidade Federal de Uberlândia Uberlândia Brazil

**Keywords:** Cerrado rupestre, endemism, Microlicieae, taxonomy

## Abstract

*Microlicia gracilis* and *Microlicia xylopodifera*, endemic to Chapada dos Veadeiros, Goiás State, Brazil, are described, illustrated and the conservation status is also provided. Both species resemble *Microlicia ordinata* and *Microlicia ramosa* that are also endemic to Goiás, by having sessile leaf with serrate and ciliate margin, pedicellate flower, triangular and short sepal and dimorphic stamens with bicolorous and polysporangiate anthers. However, *M. gracilis* differs by the long internode (2–4 mm long), concolorous, semi-amplexicaul and lanceolate leaf, and petal acuminate at the apex. *Microlicia xylopodifera* differs in having a robust xylopodium, horizontal or slightly ascending leaf and a dense crown of glandular trichomes at the apex of flower bud.

## Abstract (es)

*Microlicia gracilis* e *Microlicia xylopodifera*, endêmicas da Chapada dos Veadeiros, são descritas, ilustradas e o estado de conservação fornecido. Ambas espécies se assemelham à *Microlicia ordinata* e *Microlicia ramosa*, também endêmicas de Goiás, por apresentarem folha séssil, com margem serreado-ciliada, flor pedicelada, sépala triangular, curta e estames dimórficos com anteras bicolores e poliesporangiadas. Contudo, *M. gracilis* diferencia-se pelo entrenó longo (2–4 m compr.), folha concolor, lanceolada e semiamplexicaule, e pétala acuminada no ápice. *Microlicia xylopodifera* difere por apresentar xilopódio robusto, folha horizontal ou levemente ascendente e uma coroa densa de tricomas glandulares no ápice do botão floral.

## Introduction

*Microlicia* D.Don is a Brazilian genus with 166 species exclusive to Brazil (Flora do Brasil 2020) and with only 11 species occurring in Bolivia, Peru, Venezuela and Colombia (Renner 1993; Rull 2003; Romero 2003a; Michelangeli and Cotton 2008; Romero and Woodgyer 2015; Mendoza-Cifuentes et al. 2019; Pacifico et al. 2020a; Versiane et al. 2020). The genus reaches high diversity mainly in the campo rupestre of Bahia, Minas Gerais and Goiás (Romero 2003a, b).

The Chapada dos Veadeiros, located in the north-eastern part of the State of Goiás, is considered an important floristic component of the Cerrado biome with different phytophysiognomies at elevations that vary from 800 to 1650 metres (Munhoz and Felfili 2006; Felfili et al. 2007; Souza and Bove 2011; Romero et al. 2017). The region stands out as one of the centres of diversity of Microlicieae, being related as a recent radiation area of the tribe, due to the high number of endemic rates and high endemicity scores (Pacifico et al. 2020b). The region exhibits a significant number of endemic species of *Chaetostoma* (Silva et al. 2018), *Trembleya* (Pacifico et al. 2019) and *Microlicia* (Pilger 1903; Wurdack 1959; Diniz-Neres and Silva 2017a, 2017b; Romero et al. 2017). Moreover, at least 20 species of *Microlicia* in the State of Goiás occur in the Chapada dos Veadeiros (Naudin 1845; Cogniaux 1883; Pilger 1903; Smith 1955; Wurdack 1959; Almeda and Martins 2001; Romero et al. 2017; Diniz and Silva 2019).

*Microlicia* has been traditionally characterised in having solitary flowers with five, rarely six petals, free ovary with three or five locules and capsules with longitudinal dehiscence from the apex to the base (Almeda and Martins 2001; Romero 2003a). However, recent molecular studies show that *Microlicia*, as currently delimited, is paraphyletic and most of the morphological characters used for the circumscription of each genus in Microlicieae are homoplastic. Thus, species of *Chaetostoma* DC., *Lavoisiera* DC., *Stenodon* Naudin and *Trembleya* DC. will be included in *Microlicia*, resulting in a monophyletic genus (Versiane 2019).

In the course of preparing a taxonomic treatment of Microlicieae for the State of Goiás, some collections from Chapada dos Veadeiros could not be recognised under any name in the genus and so we concluded that these collections refer to two new undescribed species. The new species are described, compared morphologically with similar species and information about geographic distribution and conservation status is provided, as well as images of morphological structures of the two species.

## Material and methods

This study was based on the morphological analysis of specimens of *Microlicia* from the following herbaria: HEPH, HUFU, IBGE, MBM, MO, NY, UB, UEC and US (acronyms according to Thiers 2020). Specimens seen on the online platforms Reflora Virtual Herbarium (2020, https://reflora.jbrj.gov.br/reflora/herbarioVirtual), speciesLink (2020, https://www.splink.org.br/) and Tropicos (http://www.tropicos.org) were referred to here with barcode numbers. For general morphological terminology, we follow Radford et al. (1974) and the indumentum terminology follows Wurdack (1986). The leaves, colour of the petals, stamens and style were observed only in dry material. According to georeferenced data from the cited collections, the area of occupancy (AOO) and extent of occurrence (EOO) were calculated using GEOCAT (Bachman et al. 2011). The conservation status was based on the IUCN guidelines and criteria (IUCN 2019). Images of vegetative and reproductive structures were obtained using a digital camera coupled to a Zeiss stereoscopic microscope and organised on Adobe Photoshop CS6.

## Taxonomic treatment

### Microlicia gracilis

Taxon classificationPlantaeMyrtalesMelastomataceae

Fontelas & R.Romero sp. nov.

F509B553-4545-5126-8930-5BAED5C3EAE0

urn:lsid:ipni.org:names:77212346-1

Fig. 1

#### Type.

Brazil. Goiás: Alto Paraíso de Goiás, Fazenda Água Fria, ca. 10 km em direção a Teresina de Goiás, 1448 m elev., 14°04'21.7"S, 47°30'33.6"W, 27 March 2001 (fl, fr), *C. Munhoz et al. 2649* (holotype: IBGE! [IBGE00050788]; isotypes: HUFU!, MO! [MO-2024291]).

#### Diagnosis.

The new species can be recognised by the long internode (2–4 mm long), lanceolate leaf blade, attenuate and semi-amplexicaul at the base, conspicuous calyx tube (0.2 mm long) and petal acuminate at the apex.

**Figure 1.**
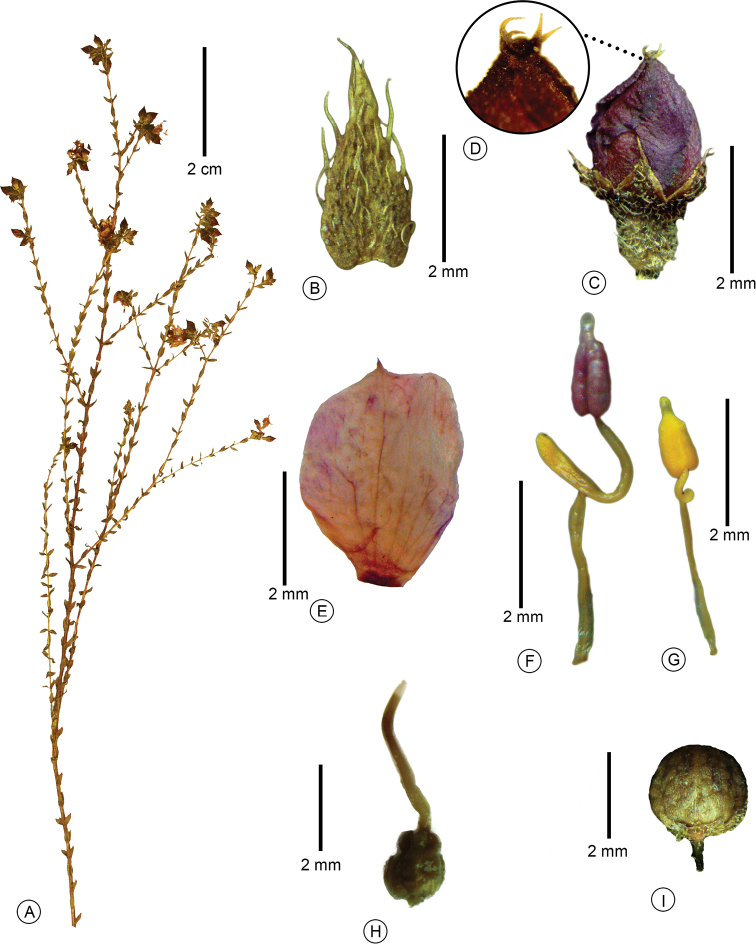
*Microlicia gracilis* Fontelas & R.Romero **A** flowering branch **B** leaf adaxial surface **C** flower bud **D** detail of the flower bud **E** petal **F** larger (antesepalous) stamen **G** smaller (antepetalous) stamen **H** gynoecium **I** closed capsule (**A–I**: *C. Munhoz et al. 1519*). Photos: Jean Fontelas.

#### Description.

Subshrub, 0.4–0.6 m tall, erect, much-branched. Stem terete, glabrous, decorticating with age. Branch fastigiate, younger branch green, quadrangular, older branch brownish, becoming terete, glabrescent and leafless with age. Branch, both surfaces of the leaf, hypanthium and sepal covered by spherical glands and setose trichomes 0.2–0.4 mm long. Leaf sessile, horizontal or ascending, lax, internode 2–4 mm long; blade 2–3 × 0.5–1 mm, concolorous, green, chartaceous, lanceolate, acute at the apex, with a terminal setose trichome ca. 0.2 mm long, base attenuate, semi-amplexicaul, margin flat, serrate, ciliate, 3-veined, usually inconspicuous on abaxial surface. Flower 5-merous, solitary, terminal or lateral, perianth actinomorphic; pedicel ca. 0.5 mm long; hypanthium 2.5–3 × 1–1.5 mm, purple or green with purple stains, urceolate, calyx tube ca. 0.2 mm long, sepal 0.5–1 × 3.5–5 mm, shorter than the length of the hypanthium, triangular, acute at the apex, with a terminal setose trichome ca. 0.2 mm long; petal 4–4.5 × 3–3.5 mm, magenta, obovate, acuminate at the apex, margin entire, glabrous; stamen 10, dimorphic, anther polysporangiate; larger (antesepalous) stamen 5, filament 2–2.5 mm long, magenta, pedoconnective 2–2.5 mm long, magenta, ventral appendage ca. 1 mm long, yellow, obtuse at the apex, anther ca. 1.8 mm long including beak, vinaceous, ovate-oblong, beak ca. 0.5 mm long; smaller (antepetalous) stamen 5, filament 2–2.5 mm long, magenta, pedoconnective 0.5–1 mm long, yellow, ventral appendage ca. 0.2 mm long, yellow, rounded at the apex, anther ca. 1.5 mm long including beak, yellow, ovate-oblong, beak ca. 0.3 mm long; ovary ca. 1.5 × 1 mm, 3-locular, pyriform, superior, glabrous; style ca. 3 mm long, magenta, terete, slightly curved; stigma punctiform. Capsule ca. 2 × 2 mm, brownish, globose, dehiscing into 3 valves from the apex, hypanthium partially covering the capsule; seed ca. 0.5 × 0.3 mm, brown, oblong, testa foveolate.

#### Distribution and habitat.

**Figure 2.**
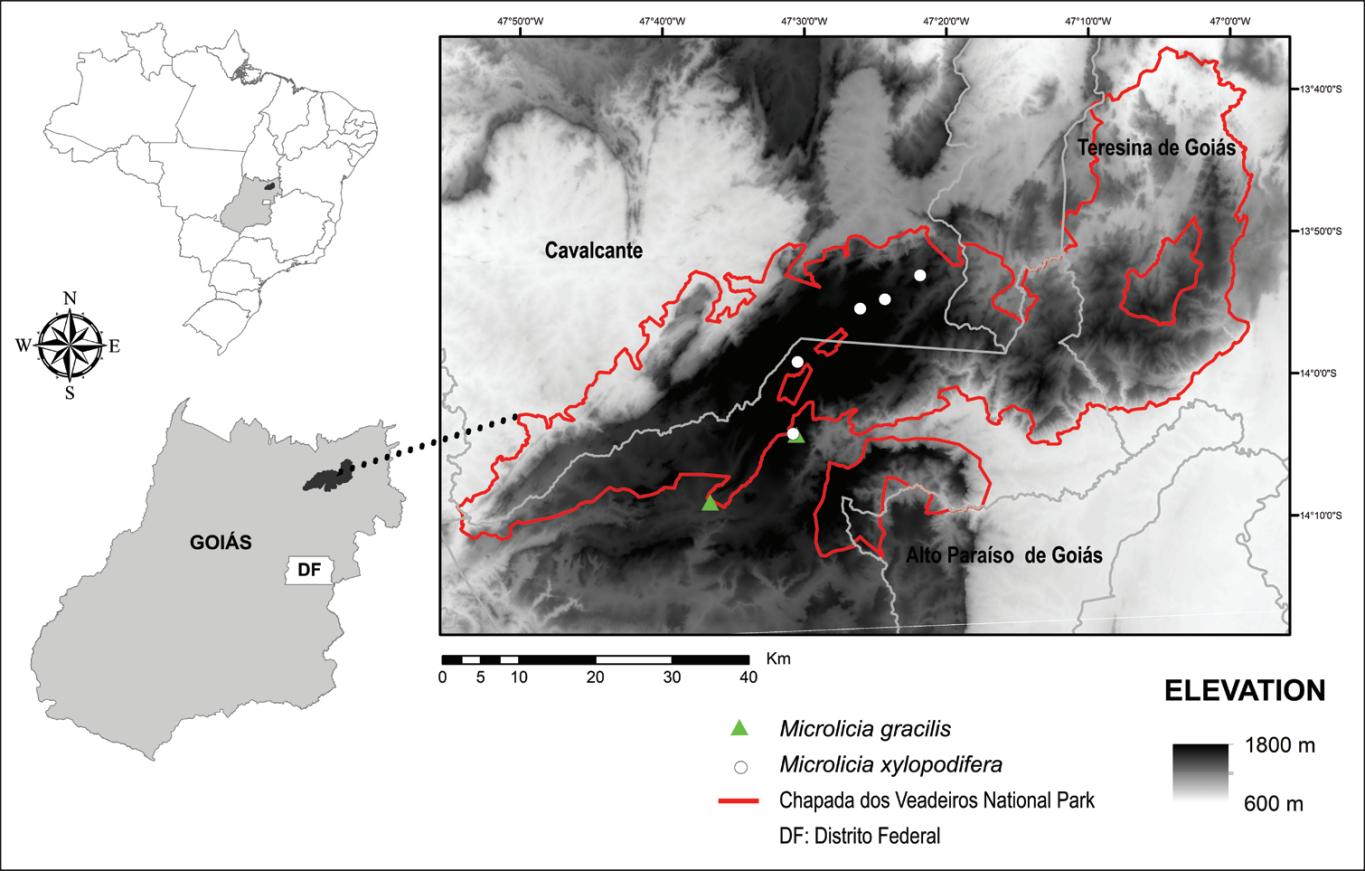
Geographical distribution of *Microlicia gracilis* and *M. xylopodifera* in the state of Goiás, Brazil.

*Microlicia gracilis* is endemic to Chapada dos Veadeiros, Goiás, Brazil, occurring in wet grasslands close to rocky outcrops and in cerrado rupestre, between 1115 m and 1448 m elevation (Fig. 2).

#### Conservation status.

*Microlicia gracilis* has a restricted extent of occurrence (EOO = 10 km^2^) and area of occupancy (AOO = 8 km^2^) and, according to the IUCN Categories and Criteria (IUCN 2019), is preliminarily assessed as Critically Endangered [CR B1ab (iii) + 2ab (iii)]. So far, collections of *M. gracilis* have been made only outside the boundaries of the Chapada dos Veadeiros National Park and, therefore, it is not protected by any conservation units. The restricted distribution of *M. gracilis* also contributes to its degree of threat, since its populations are exposed to frequent burning caused by farmers, in addition to the expansion of agricultural borders in the region (Felfili et al. 2007; Alves et al. 2013).

#### Phenology.

Flowers have been collected in March, May and June and fruits in May and June.

#### Etymology.

The specific epithet “gracilis” refers to the very delicate and fragile branches and leaves and the small size of the flowers.

#### Additional specimens examined

**(paratypes).** Brazil. Goiás: Alto Paraíso de Goiás, Fazenda Água Fria, 14°4'21"S, 47°30'33"W, 1 May 1998 (fl, fr), *R.C. Oliveira et al. 1059* (HEPH! [HEPH00020094], MBM!, UB! [UB-0110744]); Chapada dos Veadeiros, ca. 11 km da cidade, 1115 m elev., 14°09'68.5"S, 47°36'37.0"W, 16 June 1998 (fl, fr), *R. Romero et al. 5522* (HUFU!, UEC!); Fazenda Água Fria, ca. 10 km em direção à Teresina de Goiás, 1448 m elev., 14°04'21.7"S, 47°30'33.6"W, 3 June 2000 (fl, fr), *C. Munhoz et al. 1519* (HUFU!, MO! [MO-2024287]).

### Microlicia xylopodifera

Taxon classificationPlantaeMyrtalesMelastomataceae

Fontelas & R.Romero sp. nov.

555AE672-9DDE-595C-A959-1B7388E2D3C0

urn:lsid:ipni.org:names:77212347-1

Fig. 3

#### Type.

Brazil. Goiás: Alto Paraíso de Goiás, ca. 40 km N, 1250 m elev., 24 March 1971 (fl, fr), *H.S. Irwin et al. 33108* (holotype: UEC!, isotypes: CAS! [CAS0519655], NY!, US! [US-01899836]).

#### Diagnosis.

The new species can be recognised by the robust xylopodium, cespitose habit, indumentum of spherical glands, setose and glandular trichomes on branch, leaf, hypanthium and sepal and flower bud with a dense crown of glandular trichomes at the apex.

#### Description.

Subshrub, 0.1–0.2 m tall, cespitose, robust xylopodium present. Stem terete, glabrous. Branch fastigiate, younger branch green, quadrangular, older branch brownish, becoming terete, glabrescent and leafless with age. Branch, both surfaces of the leaf, hypanthium and sepal covered by spherical glands, setose and glandular trichomes 0.2–1.5 mm long. Leaf sessile, horizontal or ascending, lax, internode 2–6 mm long; blade 2.5–9 × 1.5–5.5 mm, discolorous, adaxial surface darker than the abaxial surface (in dry state), chartaceous, ovate or ovate-lanceolate, acute at the apex, with a terminal glandular trichome, 0.5–1 mm, base rounded or slightly cordate, margin flat, serrate, ciliate, 3-veined, usually inconspicuous on both surfaces. Flower 5-merous, solitary, terminal or lateral, perianth actinomorphic; pedicel 0.7–1 mm long; hypanthium 3.5–4.5 × 1.5–2 mm, green, urceolate; calyx tube ca. 0.2 mm long; sepal 1–2 × 1–1.5 mm, triangular, acute at the apex, with a terminal glandular trichome ca. 0.5 mm long; petal 5–10 × 2.5–5 mm, pink, obovate, obovate-oblong or oblong, acute at the apex, flower bud with a crown of glandular trichomes, during anthesis, the trichomes are only at the apex, margin entire; stamen 10, dimorphic, anther polysporangiate; larger (antesepalous) stamen 5, filament 2.5–3.5 mm long, pinkish, sometimes yellow, pedoconnective 2–3 mm long, pinkish, sometimes yellow; ventral appendage 1.5–2 mm long, yellow, truncate or rounded at the apex, rarely obtuse, anther 1.5–2 mm long including beak, vinaceous, ovate-oblong, beak 0.3–0.5 mm long; smaller (antepetalous) stamen 5, filament ca. 3 mm long, pinkish, sometimes yellow, pedoconnective ca. 1 mm long, pinkish, sometimes yellow, ventral appendage ca. 0.3 mm long, yellow, acute at the apex, anther ca. 1.5 mm long including beak, yellow, ovate-oblong, beak 0.3–0.5 mm long; ovary ca. 2.5 × 1.5 mm, 3-locular, pyriform, superior, glabrous; style ca. 6 mm long, pinkish, terete, slightly curved at the apex; stigma punctiform. Capsule ca. 2 × 2 mm, brown, globose, dehiscing into 3 valves from the apex, hypanthium partially covering the capsule; seed ca. 0.6 × 0.3 mm, brown, oblong, testa foveolate.

**Figure 3.**
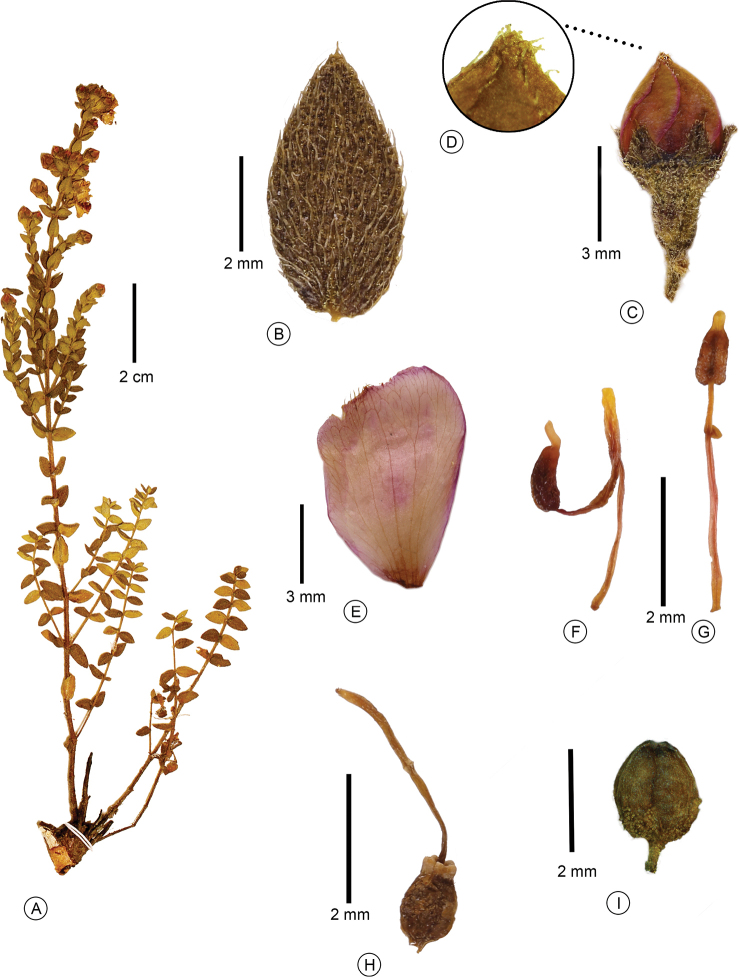
*Microlicia xylopodifera* Fontelas & R.Romero **A** habit, showing a xylopodium **B** leaf adaxial surface **C** flower bud **D** detail of the apex of the flower bud with a crown of glandular trichomes **E** petal **F** larger (antesepalous) stamen **G** smaller (antepetalous) stamen **H** gynoecium **I** closed capsule (**A–I**: *H.S. Irwin et al. 33108*). Photos: Jean Fontelas.

#### Distribution and habitat.

*Microlicia xylopodifera* is endemic to Chapada dos Veadeiros, Goiás State, Brazil, occurring in cerrado rupestre and campo limpo, on sandy soil, between 1000 m and 1800 m elevation (Fig. 2).

#### Conservation status.

*Microlicia xylopodifera* has a restricted extent of occurrence (EOO = 80 km^2^) and area of occupancy (AOO = 20 km^2^) and, therefore, we recommend that it be considered Endangered [EN B1ab (ii, iii, iv] if all IUCN (2019) guidelines are followed. So far, all populations of *M. xylopodifera* have been found inside the park boundaries, where we believe the species is protected.

#### Phenology.

Flowers and fruits have been collected in March.

#### Etymology.

The specific epithet “xylopodifera” refers to the robust xylopodium (also described as lignotubers) present in all specimens examined. Xylopodium or lignotuber is an organ that buffers the plant against extremes of water loss, temporal mineral or nutritional deficiency, providing also protection against fire (Gottsberger and Silberbauer-Gottsberger 2006).

#### Additional specimens examined

**(paratypes).** Brazil. Goiás: Alto Paraíso de Goiás, ca. 30 km ao norte da Chapada dos Veadeiros, 1000 m elev., 16 March 1969 (fl), *H.S. Irwin et al*. *24490* (NY!); ca. 19 km N, 1250 m elev., 20 March 1971 (fl), *H.S. Irwin et al*. *32788* (NY!, UEC!, US!); 8 km N, 1500 m elev., 6 March 1973 (fl), *W.R. Anderson 6433* (NY!, US!); ca. 29 km N, 800 m elev., 9 March 1973 (fl), *W.R. Anderson 6742* (NY!, US!). Teresina de Goiás, 31 km na estrada ao sul para Alto Paraíso de Goiás, 1500 m elev., 16 March 1973 (fl), *W.R. Anderson 7162* (NY!, US!).

## Discussion

Using the key to the species of *Microlicia* of the Chapada dos Veadeiros National Park proposed by Diniz and Silva (2019), *M. gracilis* and *M. xylopodifera* should be positioned close to *M. latifolia* D.O.Diniz & M.J.Silva since they have branch, leaf, hypanthium and sepal covered by setose trichomes and spherical glands, sessile and concolorous leaf blade, 2(–3)-veined, with serrate and ciliate margin, pedicellate flower, pink petal and stamens with bicolorous and polysporangiate anthers. *Microlicia latifolia* differs in having elliptic or ovate-elliptic leaf blade, apiculate at the apex, campanulate hypanthium, linear sepal, apiculate at the apex, glabrous petal and sub-isomorphic stamens with oblong anthers. Table 1 includes additional features comparing the species most similar to *M. gracilis* and *M. xylopodifera*.

**Table 1.** Comparative features of *Microlicia gracilis, M. xylopodifera* and relatives.

Characters	*M. gracilis*	*M. ordinata*	*M. ramosa*	*M. vestita*	*M. xylopodifera*
Indumentum of branch, leaf, hypanthium and sepal	Setose trichomes and spherical glands	Glandular trichomes and spherical glands	Setose trichomes and spherical glands	Setose trichomes and spherical glands	Setose, glandular trichomes and spherical glands
Leaf colour	Concolorous	Discolorous	Discolorous	Concolorous	Discolorous
Leaf base	Attenuate	Rounded or cordate	Slightly cordate, rarely rounded	Rounded or slightly cordate	Rounded or cordate
Petal apex	Acuminate	Retuse or truncate	Acute	Acute	Acute
Petal indumentum	Glabrous	Single glandular trichome at the apex	Glabrous	Single setose trichome at the apex	Glandular trichomes at the apex
Anther colours	Vinaceous and yellow	Yellow	Vinaceous and yellow	Yellow	Vinaceous and yellow
Anther, numbers of sporangia	Polysporangiate	Polysporangiate	Polysporangiate	Tetrasporangiate	Polysporangiate
References HUEG, HUFU, K, MBM, NY, UB, UEC	*C. Munhoz et al. 2649* (HUFU)	*G. & M. Hatschbach et al. 60297* (HUFU, MBM)	*J.N. Nakajima et al. 5049* (UEC, HUEG, HUFU, K, MBM, UB)	*M.L. Fonseca et al. 105* (HUFU, IBGE)	*H.S. Irwin et al. 33108* (UEC, NY)

*Microlicia gracilis* bears some resemblance to *Microlicia xylopodifera* in having sessile leaf with a lax arrangement on the branches, pedicellate flower (pedicel 0.7–1 mm long), urceolate hypanthium, dimorphic stamens with bicolorous and polysporangiate anthers. However, *M. xylopodifera* differs in having a cespitose habit, robust xylopodium and glandular trichomes covering branch, both leaf blade surfaces, hypanthium and sepal. Moreover, the apex of the flower bud is densely hairy-glandular, forming a crown at its apex and, during anthesis, the trichomes are concentrated at the apex of the petal. *Microlicia gracilis* resembles *Microlicia ramosa* Pilger, which is endemic to Goiás (Flora do Brasil 2020), for both species have branch, leaf, hypanthium and sepal covered with setose trichomes and spherical glands, sessile and ascending leaf blade, pedicellate flower, triangular sepal, magenta petal and dimorphic stamens with bicolorous and polysporangiate anthers. However, *M. ramosa* differs in having a typical branching pattern in which the branches have short secondary branches (Versiane et al. 2016), discolorous leaf (darker adaxial surface), ovate or ovate-lanceolate leaf blade with rounded or cordate base, longer pedicel (ca. 1 mm long) and the petal acute or retuse at the apex.

*Microlicia xylopodifera* is distinguished from other species of *Microlicia* by the presence of a robust xylopodium and a crown of glandular trichomes at the apex of the flower bud, which are concentrated at the apex of the petal. The new species is similar to *M. ramosa* Pilger in having sessile, discolorous and ovate or ovate-lanceolate leaves that are rounded or slightly cordate at the base, pedicellate flower, triangular sepal and dimorphic stamens with bicolorous, polysporangiate and ovate-oblong anthers. However, *M. ramosa* differs in having shorter secondary branches, 3–5-veined leaf and a vinaceous or greenish hypanthium often with vinaceous stains. *Microlicia xylopodifera* also resembles *M. ordinata* (Wurdack) Almeda & A.B.Martins, which is endemic to Goiás (Versiane et al. 2016; Machado and Romero 2020), in having branch, leaf, hypanthium and sepal covered with glandular trichomes and spherical glands, sessile and discolorous leaf, ovate-lanceolate leaf blades that are rounded or cordate at the base, triangular sepal and dimorphic stamens with bicolorous and polysporangiate anthers. However, *M. ordinata* has leaf with larger dimensions (5–20 × 2.5–13.5 mm), 3–5-veined, shorter pedicel (ca. 0.5 mm long) and petal with a single glandular trichome at the apex. In addition, the anther of the antesepalous stamen is yellow with orange stains. The new species bears some resemblance to *M. vestita* DC. which occurs in the Distrito Federal and States of Bahia, Minas Gerais, Goiás, Mato Grosso do Sul and Pará (Flora do Brasil 2020). *Microlicia vestita* also has setose trichomes and spherical glands on the branch, leaf, hypanthium and sepal, sessile leaf, ovate or ovate-lanceolate leaf blade with serrate and ciliate margin, triangular sepal and dimorphic stamens with bicolorous anthers. However, *M. vestita* differs in having imbricate leaf, denser indumentum covering the whole plant, campanulate hypanthium and tetrasporangiate anthers.

## Supplementary Material

XML Treatment for Microlicia gracilis

XML Treatment for Microlicia xylopodifera
